# Lateralization of the 5-HT_1A_ receptors in the basolateral amygdala in metabolic and anxiety responses to chronic restraint stress

**DOI:** 10.1007/s00726-023-03380-4

**Published:** 2024-02-10

**Authors:** Habib Valipour, Gholam Hossein Meftahi, Gila Pirzad Jahromi, Alireza Mohammadi

**Affiliations:** 1 Neuroscience Research Center, Baqiyatallah University of Medical Sciences, Tehran, Iran; 2 Student Research Committee, Baqiyatallah University of Medical Sciences, Tehran, Iran

**Keywords:** 5-HT_1A_ receptor, Anxiety, Basolateral Amygdala, WAY-100–635, Weight

## Abstract

Behavioral and functional studies describe hemispheric asymmetry in anxiety and metabolic behaviors in responses to stress. However, no study has reported serotonergic receptor (the 5-HT_1A_ receptor) lateralization in the basolateral amygdala (BLA) in vivo on anxiety and metabolic behaviors under stress. In the present study, the effect of unilateral and bilateral suppression of the 5-HT_1A_ receptor in the BLA on anxiety, and metabolic responses to chronic restraint stress was assessed. Male Wistar rats 7 days after cannulation into the BLA received chronic restraint stress for 14 consecutive days. 20 minutes before induction of stress, WAY-100–635 (selective 5-HT_1A_ antagonist) or sterile saline (vehicle) was administered either uni- or bi-laterally into the BLA. Behavioral (elevated plus maze; EPM, and open field test), and metabolic parameter studies were performed. Results showed that stress causes a significant increase in weight gain compared to control. In the non-stress condition, the left and bilaterally, and in the stress condition the right, left, and both sides, inhibition of 5-HT_1A_ in the BLA reduced weight gain. In the restraint stress condition, only inhibition of the 5-HT_1A_ receptor in the left BLA led to decreased food intake compared to the control group. In stress conditions, inhibition of the 5-HT_1A_ receptor on the right, left, and bilateral BLA increased water intake compared to the stress group. Inhibition of the 5-HT_1A_ receptor on the left side of the BLA by WAY-100–635 induced anxiety-like behaviors in stressed rats. Similarly, WAY-100–635 on the left BLA effectively caused anxiety-like behaviors in both EPM and open field tests in the control animals. In conclusion, it seems that 5-HT_1A_ receptors in the left BLA are more responsible for anxiety-like behaviors and metabolic changes in responses to stress.

## Introduction

Psychiatric disorders represent an enormous economic burden in modern societies (Celada et al. 2013). Anxiety disorders are the largest group of mental disorders and the leading cause of disability in most Western societies (Craske et al. 2017). Stress is a significant factor in the onset of anxiety disorders. The reciprocal association between stress and anxiety has received great attention, particularly regarding the extensive research conducted on how stress acts as a significant risk factor for anxiety disorders (Daviu et al. 2019). Changes in serotonin (5-hydroxytryptamine; 5-HT) have been introduced as a neurotransmitter that is effective in causing anxiety symptoms (Garcia-Garcia et al. 2014).

The 5-HT system plays a pivotal role in the treatment and etiology of stress-related mental illness (Harvey et al. 2004). 5-HT is synthesized in the brain and peripheral systems, which exert their actions at a broad family of receptors classified as 5-HT_1_ to 5-HT_7_. Among the types of serotonin receptors, previous studies have highlighted the role of receptor 5-HT_1A_ in the etiology of anxiety (Albert et al. 2014). 5-HT_1A_ receptors are essential factors in the psychopathology underlying stress-related psychiatric disorders, and several pharmacological treatments for mood disorders target 5-HT_1A_ receptors (Dawson and Watson 2009). There are many available drugs (such as azapirone, SSRI drugs, etc.) whose mechanism of action is through a direct or indirect effect on 5-HT receptors (Stiedl et al. 2015; Vahid–Ansari et al. 2019). Due to the mechanism of their presynaptic and postsynaptic inhibitory effects, anti-anxiety drugs reduce anxiety by activating these receptors (Schatzberg and Nemeroff 2009).

In anxiety disorder, structural and functional changes occur in some brain areas such as the hippocampus (Tang et al. 2019; Seewoo et al. 2020), the prefrontal cortex (Hare and Duman 2020), and the amygdala (Hu et al. 2022), and probably the activity of the 5-HT receptor also undergoes changes in these areas. The amygdala, especially its basolateral nucleus (BLA), plays an important role in anxiety-related behaviors. This area is noticeably responsive to stressful stimuli. It is well accepted that the amygdala is heavily innervated by serotonergic projections from the midbrain raphe nuclei, proposing that the serotonergic system is a significant mediator of neuronal activity (Bocchio et al. 2016). Studies reveal that most interneurons and pyramidal neurons in the BLA express 5-HT_1A_ receptors (Bonn et al. 2013). Infusion of 5-HT_1A_ receptor agonist into the BLA induced anxiolytic and anti-panic effects in rats (de Andrade et al. 2013), and a 5-HT_1A_ receptor antagonist suppressed the impairing impacts of stress on memory (Sardari et al. 2015).

Studies mapping the physiological roles of different amygdala regions, according to the chemical or physical damage to specific regions highlight the relationship between the amygdala and feeding management (Pineda et al. 2021). Serotonin is also an important regulator of energy intake and energy expenditure, the two inputs of energy balance. The central nervous system, serotonin is intricately involved in appetite and subsequent nutrient absorption (Yabut et al. 2019). The effects of serotonin on energy homeostasis are mediated through receptors in the hypothalamus (Jabeen Haleem 2016), while serotonin through the amygdala can modulate responses to stress and neuronal signals associated with anxiety (Bocchio et al. 2016).

Amygdala hemispheric lateralization in many behaviors and emotional processing is now well documented. This lateralization of the amygdala is often changed in patients with mental health disorders such as depression (Farahbod et al. 2010), anxiety (Hahn et al. 2011), post-traumatic stress disorder (Rauch et al. 2000), and bipolar disorder (Chepenik et al. 2010), and the pathology of these disorders are associated with abnormal amygdala function. These data propose that lateralization of the amygdala is a normal phenomenon and provides a basis for understanding the transition between acute and chronic disease states.

The high density of 5-HT_1A_ receptors in the basolateral amygdala and hemispheric asymmetry in the distribution of 5-HT_1A_ receptors (Fink et al. 2009) along with studies proposing a specific role of 5-HT_1A_ receptors in anxiety-like behaviours and emotional functioning (de Andrade Strauss et al. 2013; Morrison and Cooper 2012) leads to the question of how serotonin receptors in the left and right sides of the basolateral amygdala respond differently to anxiety-like behaviors in stressful situations. In other words, the hypothesis of this study was whether the 5-HT_1A_ receptor blockade in the BLA moderates anxiety-like behaviors following stress. Additionally, since the basolateral amygdala and 5-HT receptors are involved in metabolism, we also investigated the role of 5-HT_1A_ receptors on the left and right sides of the basolateral amygdala differently in metabolic behaviors (food intake, water intake, and weight gain) in stress and without stress conditions.

## Material and methods

### Animals

Male Wistar rats (180–250 g; age 8–10 weeks) were used in this study (n = 8 for each group). The animals were kept in groups of three in cages with a light cycle of 12/12 h, with ad libitum food and water available. The animals were placed in a temperature-controlled (22–24 °C) room. All the experiments were carried out in accordance with standard ethical guidelines and approved by the local ethics committee of the Baqiyatallah University of Medical Sciences, Tehran, Iran (approval code: IR.BMSU. REC.1397.258).

### Animals groups

As shown in the Table 1, animals were randomly divided into fourteen groups. Elevated plus maze (EPM) and open field tests were performed on different animals. Sixteen animals were used in each group (n = 16/group). Eight animals were used for the EPM assay, and eight were used for the open field and metabolic responses in each group. The stress-induced groups were restrained for 120 min for 2 weeks. Our data analysis identified no significant difference between the vehicle and control groups. Additionally, under restraint stress condition, three groups of animals were administered normal saline either on the left, right, or both sides of the BLA. The results observed in these groups did not indicate any significant difference when compared to the findings with restraint stressed animals without any injection. Therefore, we omitted all the data on vehicle groups (in with and without restraint stress conditions) for further statistical analysis to make the graphs intuitively easy to understand. Indeed, we compared all experimental groups with the control group.

**Table 1:** Experimental design and animal groups

Groups		Procedures
1	Control	Intact animals (without Cannulation surgery or normal saline injection)
2	Vehicle^RI^	Cannulation surgery + normal saline injection in the right side of the BLA
3	Vehicle^LI^	Cannulation surgery + normal saline injection in the left side of the BLA
4	Vehicle^BI^	Cannulation surgery + normal saline injection in the both sides of the BLA
5	Stress	Restraint stress (without cannulation surgery and normal saline injection)
6	Stress + Vehicle^RI^	Cannulation surgery + normal saline injection in the right side of the BLA + Stress
7	Stress + Vehicle^LI^	Cannulation surgery + normal saline injection in the left side of the BLA + Stress
8	Stress + Vehicle^BI^	Cannulation surgery + normal saline injection in the both sides of the BLA + Stress
9	WAY-100-635^RI^ without stress	Cannulation surgery + WAY-100–635 injection in the right side of the BLA (without stress)
10	WAY-100-635^LI^ without stress	Cannulation surgery + WAY-100–635 injection in the left side of the BLA (without stress)
11	WAY-100-635^BI^ without stress	Cannulation surgery + WAY-100–635 injection in the both side of the BLA (without stress)
12	WAY-100-635^RI^ + Stress	Cannulation surgery + WAY-100–635 injection in the right side of the BLA + Stress
13	WAY-100-635^LI^ + Stress	Cannulation surgery + WAY-100–635 injection in the left side of the BLA + Stress
14	WAY-100-635^BI^ + Stress	Cannulation surgery + WAY-100–635 injection in the both side of the BLA + Stress

Animals were randomly divided into eleven groups

*RI* Right side injection in the BLA, *LI* Left side injection in the BLA, *BI* Both sides injection in the BLA

### Surgical procedures and cannulation in the BLA

**Fig. 1:**
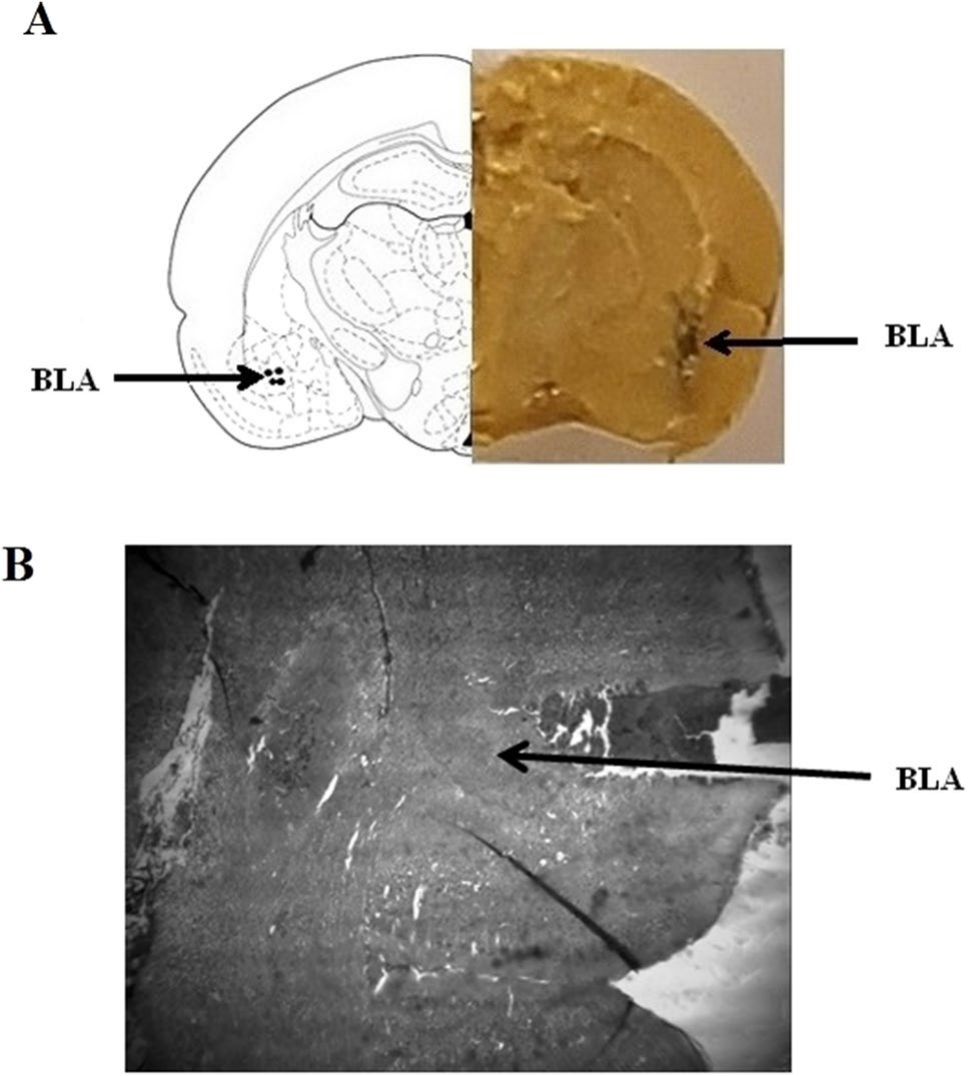
**A** A representative photomicrograph showing the injection cannula tip placements into the basolateral amygdala. **B** A representative of a hematoxylin and eosin stained showing the location of injection cannula in the basolateral amygdala

At first, the rats were intraperitoneally anesthetized with ketamine and midazolam (K: 75‐100 mg/kg + M: 4–5 mg/kg IP with supplemental doses if needed) and put in a stereotaxic apparatus (Stoelting, USA). A stainless steel guide cannula (23-gauge) was inserted into the right, left, or bilateral one mm above the BLA injection sites using the stereotaxic apparatus. The stereotaxic coordinates for BLA areas were determined by the Paxinos and Watson (2007) rat brain atlas. The coordinates for the BLA: anteroposterior (AP) = − 2.8 mm caudal to bregma, Dorsoventral (DV) = 7.5 mm ventral from the skull Surface, lateral (Lat) =  ± 4.8 mm lateral to the midline. The guide cannula was embedded in a 2 × 2 mm socket with two active and ground pins and was anchored with a steel screw and the incision was closed with dental cement. The cannula was permanently affixed to the skull surface with dental acrylic cement. After the cement was hardened, a stylet was used to close the guide cannula. The animals were brought back into a clean cage while keeping them warm after surgery (single-housed). Rats were allowed to recover for 7 days before beginning the behavioral tests. Only animals with correct cannula locations were included in the final results (Fig. 1). It should be mentioned that two animals were lost in the stress + bilateral-BLA WAY-100–635 group. Also, one animal was lost in the stress + left-BLA WAY-100–635 group. These animals were lost due to cannula displacement and replaced with new rats. For the final analysis, we used 8 animals in each group. At the end of the experiments at the verified injection site, animals were deeply anesthetized with ketamine hydrochloride (70 mg/kg, IP) + xylazine (8 mg/kg, IP), and transcardial perfusion was performed with 10% formaldehyde. The rats were decapitated and brains were removed and fixed with 10% formaldehyde. The brains were then dehydrated and paraffin-embedded blocks were prepared. Whole-brain paraffin-embedded slices, with a thickness of 5 mm, were dissected in the coronal direction on a microtome and then transferred onto glass slides. After that, the verified injection site in the BLA was identified by staining the deparaffinized sections on glass slides using hematoxylin and eosin dyes (Fig. 1B). Indeed, after our histological study, all the cannula was located in the BLA region.

### Drugs administration

WAY-100–635 (as a selective 5-HT_1A_ receptor antagonist; Cayman chemical company, Michigan 48,108 USA) or its vehicle (saline) injected via a Hamilton syringe connected to the injection cannula (dental needle head No. 30; one mm longer than guide cannula), utilizing a short piece of polyethylene tube (PE-20) and was used for all microinjections. Before testing, all drug solutions are freshly prepared. The injection needle was inserted 0.5 mm beyond the tip of the guide cannula. 20 minutes before inducing the stress, WAY-100–635 (0.1 µg in 0.1 µl/side) was administered into the left, right, and bilateral of the BLA. The other three groups were administrated WAY-100–635 into the left, right, and bilateral of the BLA without stress condition. The WAY-100–635 and saline injections were made daily for 14 consecutive days. The dosage and volume of WAY-100–635 were selected based on previous reports (de Paula and Leite-Panissi 2016). All injections were done at the speed of 0.5 µl/min and the needle was left in place for an additional two min to minimize the flow back of the solution, and the animals moved freely during this time.

### Psychological stress

In previous studies, chronic immobility (restraint) stress has been placed as psychological stress to induce depression and anxiety disorder (Chiba et al. 2012; Seewoo et al. 2020). In this study, restraint stress was obtained by putting the animal in PVC tubes with a semi-cylindrical shape (with a length of 20 cm and internal diameters of 6 cm) and a flat base with a hole in the front for breathing, which the animal was unable to move. In a continuous immobilization stress protocol, animals were stressed for 2 h/day for 2 weeks. After being restrained, rats were released back into their home cages. Control rats remained in their home cages without restraint stress procedures. Both control and restrained rats could not access food and water during the period of restraint stress exposure. The immobilization procedure took place during the light cycle from 09:00–12:00.

### Measurement of food intake, water intake, body weight

After the end of the stress, the rats were returned to their cages and 50 g of food and 50 cc of water were made available to each animal. The next day, the food intake and water intake of each rat before stress induction were measured. In addition, animals were weighed daily and changes in body weight were also assessed on the 14th day.

### Open field test

The open field is a popular animal model of anxiety-like behavior (Seibenhener and Wooten 2015). The open-field test was performed 24 h after the 14 consecutive day’s restraint stress. The open-field arena (90 cm width × 90 cm length × 40 cm height) was divided into a 3 × 3 grid of equally-sized squares using white tape. The outer section of the box was defined as the sum of all squares adjacent to a wall, not including the four corner squares. The central region of the box is only included in the middle square. The test started by placing a rat on the same side of the outer section such that the rat could visit the center area first or move to one of the corners. The behavior of each rat in the open-field box was recorded on video and scored manually. The following behaviors were recorded for each animal for 5 min: time spent in the center zone and distance traveling, stretched-attend posture (stretching forward with the forelimbs extended, often with the back arched to maintain a low profile), rearing (standing on hind legs, with or without contact with the sides of the arena), grooming (using paws or tongue to clean/scratch body). Finally, the total distance traveled during the 5 min of the test was calculated to examine locomotor changes. In this test, decreased rearing, grooming, and stretching are considered signs of increased anxiety. Also, in the open field test, the increase in time spent in the central area of the test is considered a sign of less anxiety.

### Elevated plus maze (EPM)

The elevated plus maze has been used to measure the anxiety-related behavior of rodents (Walf and Frye 2007). The EPM test was performed 24 h after the 14 consecutive day’s restraint stress. We used a maze that consisted of four arms with a length of 50 cm, the two closed arms were closed with 40 cm side walls, and the two open arms had a 2 cm protector. The intersection of four components was placed on a 100 cm base. Briefly, rats are placed at the junction of the four arms of the maze, facing an open arm and closed arm, and a video-tracking system for 5 min records entries/duration in each arm. An increase in open-arm activity (duration or entries) reflects anti-anxiety behavior. The distance traveled in the closed arm was used to examine locomotor changes. In the EPM test, reducing the number of rearing and stretching is considered anxiety, and increasing the time spent and the number of entries into the open arms reduces anxiety symptoms.

### Statistical analysis

Data were expressed as mean ± standard error (Mean ± SEM) for eight animals in each group.

Two-way analysis of variance (Two-way-ANOVA) was applied using WAY-100–635 and psychological stress as factors, followed by the Tukey post hoc test. Differences with p < 0.05 were considered statistically significant. The results of the Two-way ANOVA for the main effects of WAY-100–635, psychological stress and the WAY-100–635 × psychological interaction, and post hoc tests are reported to analyze the difference between other groups in the dependent variable (for example, weight, food, time, etc.). All data were analyzed using IBM SPSS statistics (version 26).

## Results

### ***Effects of 5-HT***_***1A***_*** suppression in the BLA on stress-induced alteration in weight gain, food and water intake***

### Weight gain

A two-way ANOVA was conducted that examined the effect of WAY-100–635 and psychological stress on weight gain. The effect of WAY-100–635 and psychological stress on weight gain was significant, but their interaction effect was not significant [(F(7,64) = 13.367, p = 0.001), (WAY-100–635 effect, F = 10.550, p = 0.0001; psychological stress effect, F = 12.060, p = 0.0001; interaction of WAY-100–635 × psychological stress, F = 1.717, p = 0.189].

**Fig. 2:**
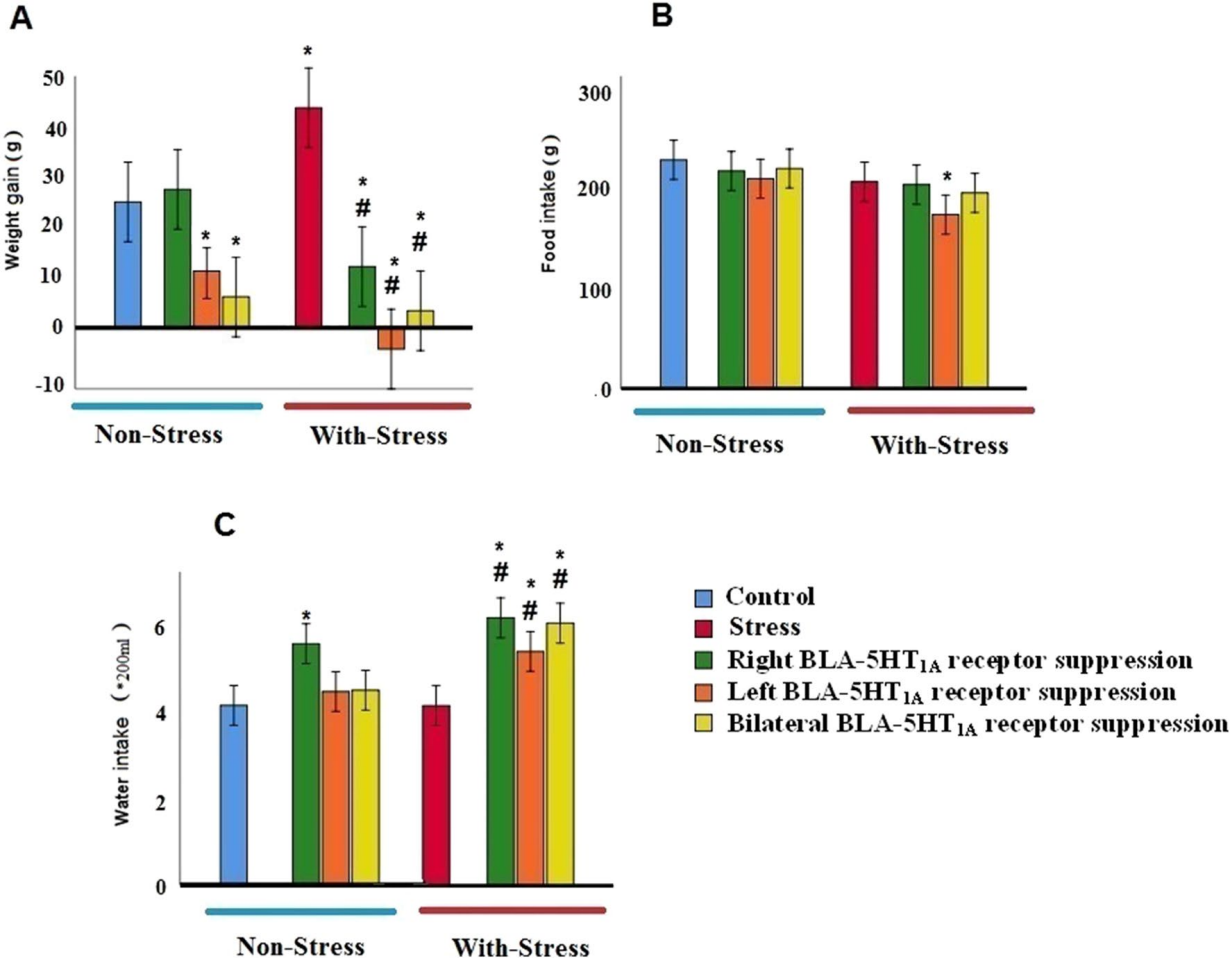
Effect (mean ± SEM) of the intra-BLA administration of WAY-100–635 (selective 5-HT_1A_ antagonist) and induced chronic restraint stress on weight gain (**A**), on food intake (**B**) and on water intake (**C**) on day 14th (n = 8). *p < 0.05 compared to the control group. #p < 0.05 compared to the stress group

As shown in Fig. 2A, the post-hoc test showed that there was a significant difference in weight changes between different groups. In non-stress conditions, the right-side administration of WAY-100–635 into BLA did not show a significant difference in comparison with the control group. But the left and bilateral injection of WAY-100–635 into BLA reduced the weight gain compared to the control group. The post-hoc test showed that stress causes a significant increase in weight gain when compared to a control (p < 0.01). The administration of WAY-100–635 and the inhibition of 5-HT_1A_ on the right, left and both sides of the BLA in the stress condition led to the reduction in weight gain when compared to the stress group.

### Food intake

Two-way ANOVA showed that the effect of psychological stress on food intake was significant, but the effect of WAY-100–635 and their interaction effect were not significant [(F(7,64) = 2.985, p = 0.010), (WAY-100–635 effect, F = 2.156, p = 0.125; psychological stress effect, F = 9.565, p = 0.003; interaction of WAY-100–635 × psychological stress, F = 0.652, p = 0.525)].

Post-hoc test results indicated that there was no statistically significant (p < 0.05) difference between the control and the stress groups in food intake. Also, inhibition of the 5-HT_1A_ receptor in the left and/or right or both sides of the BLA in non-stress conditions did not significantly change food intake than the control‏ group. In the restraint stress condition, the administration of WAY-100–635 and the inhibition of the 5-HT_1A_ receptor in the left BLA led to a decrease in food intake compared to the control group (Fig. 2B). Nevertheless, the right and both sides administrations of WAY-100–635 to the BLA in stress conditions didn’t change food intake relative to the control‏ and stress groups.

### Water intake

The statistical analysis showed that there was no significant difference between stress and control groups in the water intake. As shown in Fig. 2C, in non-stress conditions, only the right-BLA WAY-100–635 treated animals exhibited a significant increase in water intake compared to the control animals. The post-hoc test showed that in stress-induced groups, administration of WAY-100–635 in the right, left, and bilateral BLA significantly increased water intake compared to the stress and control groups [(F(7,64) = 13.602, p = 0.001). (WAY-100–635 effect, F = 8.600, p = 0.001; psychological stress effect, F = 31.747, p = 0.0001; interaction of WAY-100–635 × psychological stress, F = 2.211, p = 0.119; Two-way ANOVA revealed that the effect of WAY-100–635 on water intake was significant, but the effect of psychological stress and their interaction effect was not significant].

### ***The effects of inactivation of the 5-HT***_***1A***_*** receptor in the BLA on anxiety-like behaviors in the EPM test in stress***

**Fig. 3:**
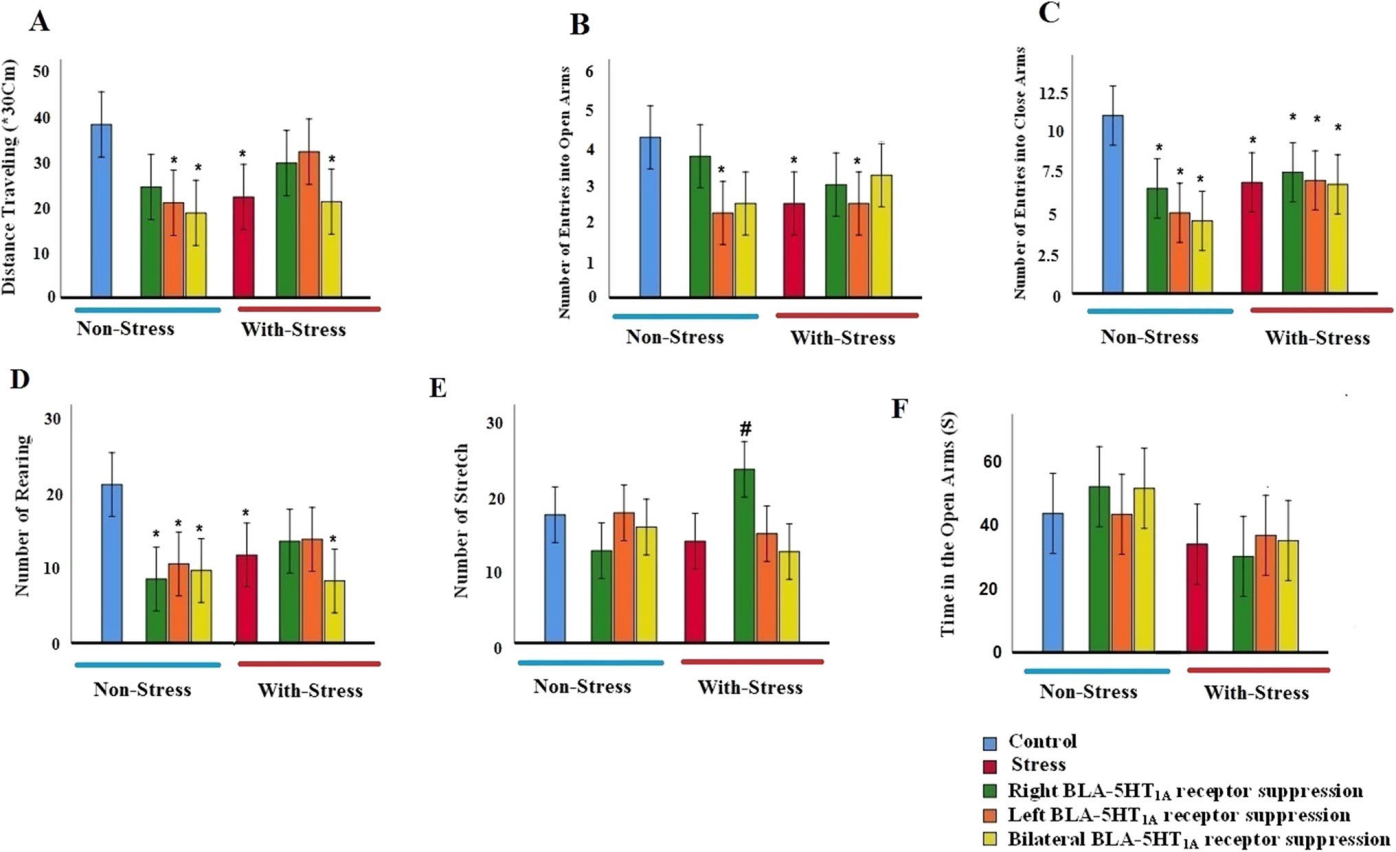
Influence of intra-BLA micro-injection of WAY-100–635 prior to chronic restraint stress on the elevated plus maze test in the rat. **A** Distance traveling, **B** the number of entries into the open arms and **C** the number of entries into the closed arms, **D** the number of rearing, **E** the number of stretches, and **F** time spent in the open arms within 5 min. The data are expressed as the mean ± S.E.M. for 8rat/group. *p < 0.05 as compared to the control group, #p < 0.05 as compared to the stress

As shown in Fig. 3A, stress (stress group) causes a significant (p < 0.01) decrease in the distance traveling when compared to the control group. In non-stress conditions, the left and bilateral administration of WAY-100–635 into the BLA significantly reduced distance traveling in comparison with the control group. But, when WAY-100–635 was administrated into the right BLA there was no significant difference in the distance traveling compared to the control group. In the restraint stress condition, the post-hoc test showed that only bilateral suppression of the 5-HT_1A_ receptors in the BLA reduced distance traveling in comparison with the control group in the EPM test. Nevertheless, left or right microinjection of WAY-100–635 to the BLA in stressed rats didn’t change the distance traveling relative to the control ‏group [(F (7,64) = 3.536, p = 0.003) (WAY-100–635 effect, F = 2.450, p = 0.096; psychological stress effect, F = 4.659, p = 0.035; interaction of WAY-100–635 × psychological stress, F = 0.775, p = 0.465; Two-way ANOVA, effect of psychological stress on distance traveling was significant, the effect of WAY-100635 and their interaction effect was not significant].

Restraint stress (stress group) statistically decreased the number of entries into the open arms when compared to the control group. Animals receiving the WAY-100–635 injection into the left BLA in both non-stress and restraint stress conditions had a significant reduction in the number of entries into the open arms when compared to the control group. The right or both sides administrations of WAY-100–635 into BLA in the stressed rats did not show a significant difference in comparison with the control animals (Fig. 3B) [(F(7,64) = 2.872, p = 0.012) (WAY-100–635 effect, F = 2.872, p = 0.065; psychological stress effect, F = 0.060, p = 0.888; interaction of WAY-100–635 × psychological stress, F = 1.675, p = 0.197); Two-way ANOVA, the effect of WAY-100–635, psychological stress and their interaction effect on number of entries into open arms was not significant]. As shown in Fig. 3C, restraint stress (stress group) significantly reduced the number of entries into the closed arms when compared to the control group. Also, the left, right or bilateral microinjection of WAY-100–635 into the BLA groups showed a reduction in the number of entries into the close arms than the control group. Thus, inhibition of 5-HT_1A_ in the BLA did not prevent a reduction in the number of entries into the closed arms after the stress [(F(7,64) = 4.594, p = 0.001) (WAY-100–635 effect, F = 1.216, p = 0.304; psychological stress effect, F = 5.527, p = 0.020; interaction of WAY-100–635 × psychological stress, F = 0.770, p = 0.263)].

As shown in Fig. 3D, restraint stress (stress group) causes a significant decrease in the number of rearing when compared to the control group. Similarly, in the non-stress condition, the microinjection of WAY-100–635 and the inhibition of 5-HT_1A_ receptors on the left, right, and both sides of the BLA led to decreases in rearing relative to the control group. In stressed rats, only bilateral microinjection of WAY-100–635 reduced rearing than the control group [(F(7,64) = 3.941, p = 0.001) (WAY-100–635 effect, F = 1.180, p = 0.315; psychological stress effect, F = 1.778, p = 0.188; interaction of WAY-100–635 × psychological stress, F = 1.224, p = 0.302].

Figure 3E demonstrates the stretch behavior within 5 min by the rat in the EPM. Restraint stress did not change the stretch in the open arms when compared to the control group. The animals receiving the WAY-100–635 injection in the non-stress condition had no significant changes in the number of stretches when compared to the control and stress groups. The left or both sides administrations of WAY-100–635 into BLA in the stressed rats did not show a significant difference in comparison with the control and stress‏ animals. But the right injection of WAY-100–635 into BLA showed that restraint stress could increase the number of stretches compared to the stress animals [(F(7,64) = 3.738, p = 0.002) (WAY-100–635 effect, F = 2.229, p = 0.117; psychological stress effect, F = 1.113, p = 0.296; interaction of WAY-100–635 × psychological stress, F = 9.335, p = 0.0001)]. No significant difference in total time spent in the open arms was observed between all groups (Fig. 3F).

### ***The effects of inactivation of the 5-HT***_***1A***_*** receptor in the BLA in the open field test***

**Fig. 4:**
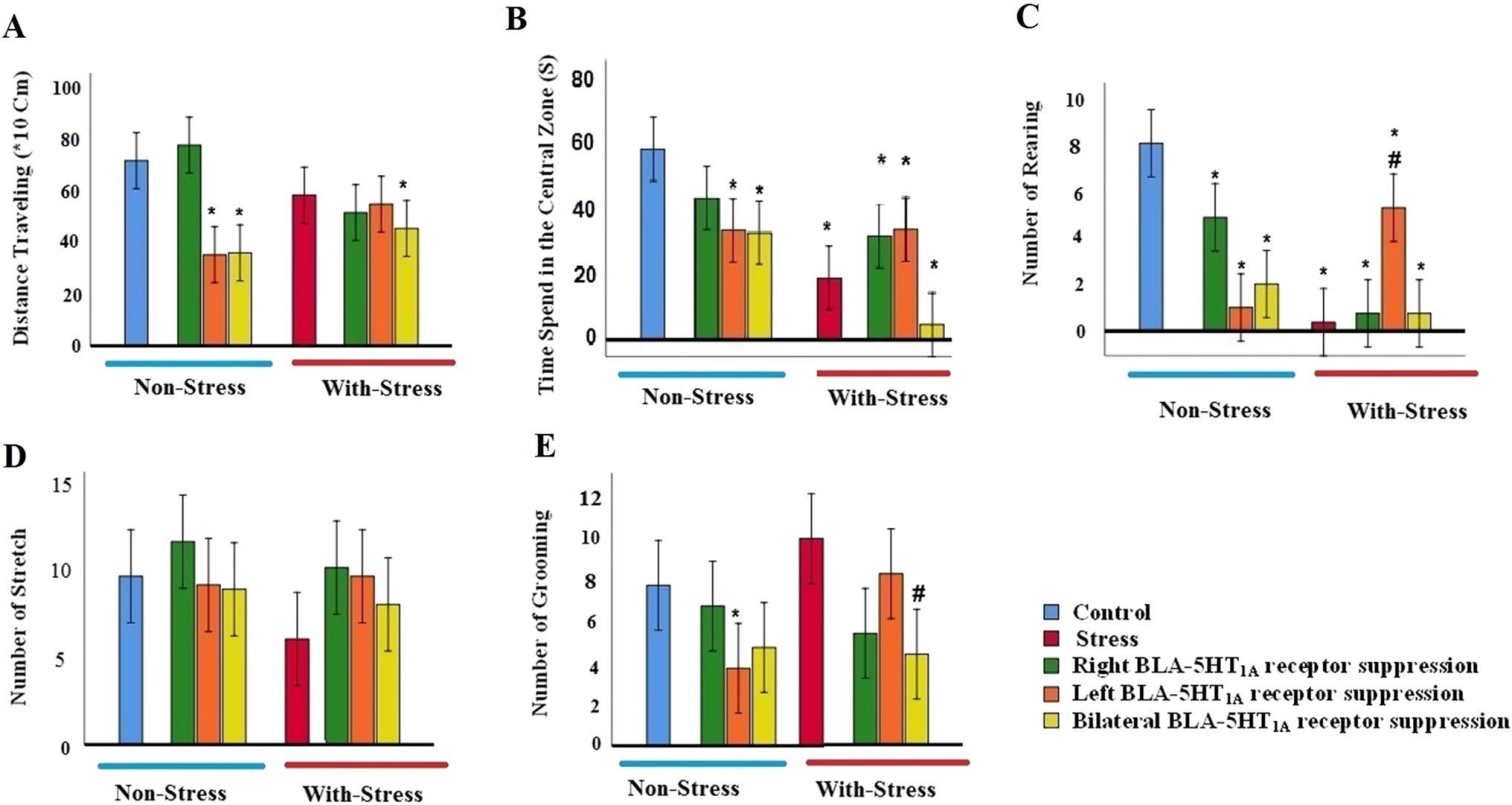
Effects of intra-BLA infusion of WAY-100–635 prior to chronic restraint stress in the anxiety-like behavior in the open field test. **A** Distance traveling, **B** the time spent in the central area, **C** the number of rearing, **D** the number of stretches, **E** the number of grooming behaviors in the open field test in the different groups. Data expressed as Mean ± S.E.M. *p < 0.05 as compared to the control group, #p < 0.05 as compared to the stress

We further investigated the effects of the 5-HT_1A_ receptor in the BLA on anxiety-like behavior by employing a circular open field 24 h after the 14 consecutive day’s restraint stress. Two-way ANOVA followed by Tukey’s analysis of distance traveling revealed no significant differences between control and after the chronic stress procedure (stress group) (Fig. 4A). In non-stress conditions, the left and bilateral inhibition of 5-HT_1A_ receptors in the BLA significantly reduced distance traveling in comparison with the control group in the open field test. However, when WAY-100–635 was administrated into the right BLA there was no significant difference in the distance traveling compared to the control group in the open field test. Results showed that inhibition of 5-HT_1A_ receptors in the left or right side of the BLA in stressed rats didn’t significantly change the distance traveling compared to the control‏ group. But, the administration of WAY-100–635 and the inhibition of 5-HT_1A_ receptors on both sides of the BLA led to a decrease in the distance traveling relative to the control group [(F_(7,64)_ = 8.068, p = 0.001, (WAY-100–635 effect, F = 11.136, p = 0.0001; psychological stress effect, F = 0.048, p = 0.828; interaction of WAY-100–635 × psychological stress, F = 9.870, p = 0.0001); effect of WAY-100–635 and interaction between WAY-100–635 and psychological stress on distance traveling was significant, the effect of psychological stress was not significant].

Post-hoc test showed that in the stressed animals (stress group) the time spent in the central zone of the open field significantly decreased compared to the control group. Also, in non-stress conditions, microinjections of WAY-100–635 into the left and both sides of the BLA reduced the time spent in the central zone more than the control group. However, in the non-stress condition, microinjections of WAY-100–635 into the right and the left BLA increased the time spent in the central zone more than the stress group. As shown in Fig. 4B in stress condition, the left, right and bilateral inhibition of 5-HT_1A_ receptors in the BLA significantly reduced the time spent in the central zone in comparison with the control group in the open field test [(F(7,64) = 10.624, p = 0.001). (WAY-100–635 effect, F = 8.434, p = 0.001; psychological stress effect, F = 11.069, p = 0.002; interaction of WAY-100–635 × psychological stress, F = 4.316, p = 0.018)].

Restraint stress (stress group) statistically decreased the number of rearing behaviors relative to the control group in the open field test. Also, animals receiving the WAY-100–635 injection into the left, right and both sides of the BLA in non-stress conditions had a significant reduction in rearing in the open field compared to the control group. In the restraint stress, only inhibition of 5-HT_1A_ in the left BLA increased the rearing in comparison with the stress animals (Fig. 4C). (F(7,64) = 15.895, p = 0.001) (WAY-100–635 effect, F = 3.715, p = 0.065; psychological stress effect, F = 0.731, p = 0.396; interaction of WAY-100–635 × psychological stress, F = 19.063, p = 0.001).

No significant difference in the number of stretches in the open field test was observed between all groups in stress and without stress conditions (Fig. 4D). The results revealed that in non-stressed animals, microinjections of WAY-100–635 into the left BLA reduced grooming when compared to the control group. In the stressed rats, only bilateral microinjection of WAY-100–635 reduced grooming than the stress [(F(7,64) = 3.941, p = 0.001; WAY-100–635 effect, F = 1.180, p = 0.315; psychological stress effect, F = 1.778, p = 0.188; interaction of WAY-100–635 × psychological stress, F = 1.224, p = 0.302)] (Fig. 4E).

## Discussion

Despite many studies that have investigated the role of serotonin receptors in the BLA in stress responses, there is a lack of studies on the differences between the left and right sides of 5-HT_1A_ receptors in the BLA in stress responses. Thus, in the present study, we investigated the roles of the right and the left sides of 5-HT_1A_ receptors in the BLA in metabolic parameters, and anxiety-like behavior changes in response to chronic immobilization stress. For this purpose, the chronic restraint stress method was used to induce anxiety-like behaviors and we suppressed the 5-HT_1A_ receptor in the BLA using WAY-100–635, which is an antagonist of the 5-HT_1A_ receptor, unilaterally (left–right) and bilaterally. The dosage and volume of WAY-100–635 were selected based on previous reports (de Paula and Leite-Panissi 2016).

The results demonstrated that the animal weight in stressed animals was significantly increased in comparison with the control group. However, there was no significant difference in food and water intake in the control and stress group animals. Ample evidence reported that psychological and chronic stress leads to increased obesity and increased food consumption in animal models. Studies suggested that this increase is due to the body’s homeostatic response to stress caused by allostatic load (Dimitratos et al. 2021). The need to regulate the body’s energy in stress conditions (fight and flight response) is introduced as an effective factor in weight changes. Secretion of glucocorticoids in response to activating the hypothalamus–pituitary–adrenal (HPA) axis acts as a regulator of the body’s metabolic activities and energy storage (Kuckuck et al. 2023). Long-term activation of the HPA axis may lead to an increase in abdominal visceral fat aggregation and weight gain through enhanced cortisol secretion (Gianotti et al. 2021). In line with these studies, in the present study, stress caused weight gain in animals. Therefore, this weight gain in stressed animals may have happened due to their increased need for energy and regulation of body homeostasis.

In our study, in non-stressed rats results showed that the left and bilateral inhibition 5-HT_1A_ receptor in the BLA, and in stress rats the left, right and bilateral inhibition decreases weight gain more than the stress group. Food intake was reduced only when 5-HT_1A_ receptors in the left BLA were inhibited in stressed animals, however, inhibition of the 5-HT_1A_ receptor in the right, left, and bilateral sides of the BLA in stress-induced groups enhanced water intake compared to the stress and control groups. On the other hand, 5-HT_1A_ receptor suppression in the BLA could modulate the weight gain induced by restraint stress. According to our knowledge, no studies have investigated the 5-HT_1A_ receptor lateralization in the BLA region on metabolic parameters. The amygdala, especially its BLA portion, is involved in controlling nutritional behavior like food preferences (Zamyad et al. 2021), as well as predicting susceptibility to weight gain in males and females (Sun et al. 2015). Several lines of evidence show the 5-HT_1A_ receptor is involved in physiological functions such as control of energy balance (Mavanji et al. 2022). In this regard, some previous studies showed that both the full 5-HT_1A_ receptor agonist 8-OH-DPAT (8-hydroxy-2-(di-n-propylamino) tetralin) and selective partial agonist ipsapirone could enhance food intake in rodents (De Vry and Schreiber 2000; Ebenezer et al. 2007). Also, the hyperphagia induced by the 5-HT_1A_ receptor agonist in mice could be reversed by co-administration of the WAY-100–635 (Ebenezer et al. 2007). Dill et al. (2013) revealed that 5-HT_1A_ receptor antagonists inhibit feeding by reducing the number of meals, and induce weight loss without an aversive side effect. Yadav et al. 2011 indicated that selective 5-HT_1A_ receptor antagonist decreased twenty-four h food intake in leptin-deficient ob/ob or chow-fed WT mice when administered alone. Also, they showed that mice in which the 5-HT_1A_ receptor was specifically knocked out from proopiomelanocortin (POMC) neurons in the arcuate nucleus did not show anorexia, suggesting that appetite regulation is directly modulated via 5-HT_1A_ expression in these hypothalamic neurons. Furthermore, the amygdala appears to play an important role in the hedonic aspects of eating (van Galen et al. 2021) and, thus, it is possible that suppression of the 5-HT_1A_ receptor in the BLA region promotes weight loss by reducing the pleasure of eating and appetite.

The behavioral tests, EPM, and open field test, 24 h after 14 consecutive restraint stresses were investigated. The present observations have shown that the restraint stress paradigm is anxiogenic, as reflected by the significant reduction in open-arm activity (reduces the spent time in the central zone of the open field) concomitant with an increase in distance traveled, in the anxiety index in the EPM. Our data analysis in the open field test, where a significant decrease in the time spent in the central zone of the open field and rearing behaviors were detected in chronic restraint stress-exposed relative to control animals, supports the EPM observations. A relationship between stress and anxiety was found in several studies. For instance, studies have revealed chronic restraint stress-induced anxiety-like behaviors in rodents, consistent with our results. Moreno-Martínez et al. (2022) propose that chronic restraint stress-induced anxiety-like behavior might be accounted for by a decrease in synaptic connectivity of the central nucleus of the amygdala.

The BLA plays a pivotal role in anxiety disorders and is extremely sensitive to stress. There is a close relationship between chronic stress-induced anxiety and neuroplasticity changes in the BLA (Moreno-Martínez et al. 2022). Several lines of evidence show that chronic stress leads to the hyperexcitability of principal output neurons in the BLA, thereby promoting anxiety, by decreasing afterhyperpolarization in rats that are sensitive to stress (Sharp 2017). Also, Liu et al. (2020) showed a target cell-based dysregulation of dorsomedial prefrontal cortex-to-BLA transmission for chronic restraint stress-induced anxiety.

The behavioral differences observed in the EPM (such as the decreased the distance traveled, the decreased number of entries in the opened arms, the number of entries in the enclosed arms, and the number of rearings) may be due to an impairment in the locomotors activity. In line with our study, several investigations showed that chronic restraint stress is linked with decreased locomotor activity. For instance, Bassey et al. (2023) revealed that female rats reduced locomotor activity in response to acute and chronic restraint stress. Also, Upadhyaya et al. (2022) exhibited chronic restraint stress in rats, reduced locomotor activity. In the elevated EPM test, bilateral administration of WAY-100–635 into the BLA showed a significant reduction in locomotor activity (reduced distance traveled, number of entries into the closed arms, and rearing) compared with control rats. The reduction in locomotor activity was observed with a decrease in the density of 5-HT_1A_ receptors in the medial hypothalamus (Li et al. 2004). There is a negative correlation between the reduction in time spent in the open arms of EPM and the density of 5-HT_1A_ receptors located in the central amygdala. The density of 5-HT_2C_ receptors in the basolateral nucleus of the amygdala showed a significant correlation with both reduced time spent and distance traveled in the open arms of EPM (Li et al. 2012). However, there is little evidence that the BLA 5-HT_1A_ receptors directly control motor function. Additional research will be necessary to directly assess this hypothesis.

There is considerable interest in the role of 5-HT_1A_ receptors in the expression of anxiety behaviors. Enhanced neuronal signaling at 5-HT_1A_ receptors is related to decreased anxiety. For instance, 5-HT_1A_ partial receptor agonists like buspirone are administrated clinically for their anxiolytic effects (Hindmarch et al.^1992^). Transgenic mice overexpressing the 5-HT_1A_ receptor indicate reduced anxiety-like behavior compared to wild-type mice (Kusserow et al. 2004). Furthermore, 5-HT_1A_ knockout mice exhibited higher levels of anxiety-like behaviors than controls (Akimova et al.^2009^). Neuroimaging investigation revealed that people with low 5-HT_1A_ binding are more likely to exhibit clinical anxiety and have elevated basal cortisol levels (Lanzenberger et al. 2010). Although previous studies have shown that 5-HT_1A_ receptor agonists enhance anxiolytic effects (Li et al.^2012^), other studies suggest that postsynaptic suppression of this receptor causes anxiolytic effects. For instance, Rombolà et al. (2020) showed that the 5-HT_1_ receptor plays a crucial role in reducing anxiety. They indicated that WAY-100–635 reduces anxiety-like behaviors of subjects in the open field and EPM tests. Also, Schreiber and De Vry (1993) reported that postsynaptic antagonism of the 5-HT_1A_ receptor leads to anxiolytic effects, while presynaptic antagonism (in the raphe nucleus) leads to anxiogenic effects. In addition, another study proposes an anxiolytic-like impact of the WAY-100–635 on non-human primates (Barros et al. 2003). In the present study, the results showed that suppression of the 5-HT_1A_ receptor in the BLA induces anxiety-like behaviors with and without stress conditions.

Evidences display that serotonin receptors in the BLA are involved in anxiety behaviors. In this regard, Bocchio and colleagues (2016) showed that the BLA plays a central role in response to fearful stimuli. Moreover, Sengupta et al. (2017) emphasized the critical role of the amygdala's serotonin receptors (5-HT_1A_ and 5-HT_2A_) in the emotion process. Studies propose that pharmacological activation of BLA 5-HT_1A_ receptors reduces anxiety and fear when responses are conditioned. For instance, infusion of a selective 5-HT_1A_ receptor agonist into the BLA decreases fear-potentiated startles (Groenink et al. 2000), fear conditioning (Li et al. 2006) and inhibitory avoidance in the elevated T-maze (Zangrossi et al.^1999^).

Many studies have shown hemisphere asymmetry. Greater right hemispheric activation in the amygdala is found to be associated with increased anxiety in rats (Mundorf and Ocklenburg 2023). It appears that the left and right amygdala perform separate functions in the emotion regulation process, as indicated by the differing modulation of mediofrontal cortical functional connectivity. This discrepancy can be seen as a potential vulnerability marker for susceptibility to stress and pathologic anxiety due to harm avoidance (Baeken et al. 2014). García-García et al. (2020) proposed that emotional context alters food and nonfood perception, and the left amygdala seems to play a crucial role in mediating these effects.

The present results revealed that in non-stress conditions, inhibition of 5-HT_1A_ receptors in the left and bilateral sides of the BLA reduced distance traveling and grooming in both EPM and open field tests. Moreover, in the open field test in non-stressed rats, the inhibition of 5-HT_1A_ receptors on the left and both sides of the BLA led to decreases in rearing. The present results demonstrated that suppression of the 5-HT_1A_ receptor in the BLA induced psychiatric symptoms in both stress and non-stress conditions. Also, results revealed that suppressing the 5-HT_1A_ receptor in the left BLA (in the left and bilateral sides of the BLA reduced distance traveling, rearing and grooming) is more responsible for anxiety-like behavior compared to the right BLA. Lateralization of 5-HT_1A_ serotonin receptor function in the amygdala in processing emotional stimuli has been observed in some previous studies (Selvaraj et al. 2018). Moreover, some previous studies have pointed to asymmetry in the distribution of the serotonin transporter in the hemispheres (Kranz et al. 2014). Andersen and Teicher, (1999) revealed that higher serotonin concentrations in the right amygdala were associated with higher anxiety, whereas expression of the serotonin transporter upon drug infusion was shown to be asymmetric (Tellez et al. 2010). Fink et al. (2009) indicated strong asymmetry of the human 5-HT_1A_ receptor in the areas of auditory and language processing. Therefore, these studies suggest an asymmetric serotonergic system and related functions. In line with these studies, the results of the present study showed the laterality of the 5-HT_1A_ receptor in the left BLA region in anxiety-like behaviors. To the best of our knowledge, no study described the different hemispheric functions of 5-HT_1A_ receptors in the BLA in the chronic restraint stress condition. The present study supports the view of asymmetric 5-HT_1A_ receptors in the BLA on anxiety and metabolic alternation induced by stress.

One limitation of the present study is that previous investigations have indicated that WAY-100–635 exhibits potent agonism at the D4 receptor (Marona-Lewicka and Nichols 2011), implying that its influence on anxiety-like behaviors in the BLA could be partially mediated by its effects on D4 receptors.

## Conclusion

In conclusion, the results of the present study using the chronic restraint stress paradigm in rats provided more evidence of chronic stress-induced anxiogenic effects and changes in metabolic behaviors. Also, suppression of the 5-HT_1A_ receptor in the BLA region leads to an increase in anxiety-like behaviors and metabolic changes. According to these results, 5-HT_1A_ receptors in the left BLA are more responsible for anxiety-like behaviors and metabolic changes in responses to stress. Thus, 5-HT_1A_ receptors in the BLA may play a functional role in the neural circuitry mediating chronic stress-induced metabolic changes and anxiety-like behavior.

## Data Availability

The data that support the findings of this study are available from the corresponding author, upon reasonable request.
